# Disseminating quality improvement: study protocol for a large cluster-randomized trial

**DOI:** 10.1186/1748-5908-6-44

**Published:** 2011-04-27

**Authors:** Andrew R Quanbeck, David H Gustafson, James H Ford, Alice Pulvermacher, Michael T French, K John McConnell, Dennis McCarty

**Affiliations:** 1 Center for Health Enhancement Systems Studies, Industrial and Systems Engineering Department, University of Wisconsin-Madison, Madison, WI 53706, USA; 2 Departments of Sociology, Epidemiology and Public Health, and Economics, University of Miami, Coral Gables, FL 33124, USA; 3 Department of Emergency Medicine, Oregon Health and Science University, Portland, OR 97239, USA; 4 Department of Public Health and Preventive Medicine, Oregon Health and Science University, Portland, OR 97239, USA

## Abstract

**Background:**

Dissemination is a critical facet of implementing quality improvement in organizations. As a field, addiction treatment has produced effective interventions but disseminated them slowly and reached only a fraction of people needing treatment. This study investigates four methods of disseminating quality improvement (QI) to addiction treatment programs in the U.S. It is, to our knowledge, the largest study of organizational change ever conducted in healthcare. The trial seeks to determine the most cost-effective method of disseminating quality improvement in addiction treatment.

**Methods:**

The study is evaluating the costs and effectiveness of different QI approaches by randomizing 201 addiction-treatment programs to four interventions. Each intervention used a web-based learning kit plus monthly phone calls, coaching, face-to-face meetings, or the combination of all three. Effectiveness is defined as reducing waiting time (days between first contact and treatment), increasing program admissions, and increasing continuation in treatment. Opportunity costs will be estimated for the resources associated with providing the services.

**Outcomes:**

The study has three primary outcomes: waiting time, annual program admissions, and continuation in treatment. Secondary outcomes include: voluntary employee turnover, treatment completion, and operating margin. We are also seeking to understand the role of mediators, moderators, and other factors related to an organization's success in making changes.

**Analysis:**

We are fitting a mixed-effect regression model to each program's average monthly waiting time and continuation rates (based on aggregated client records), including terms to isolate state and intervention effects. Admissions to treatment are aggregated to a yearly level to compensate for seasonality. We will order the interventions by cost to compare them pair-wise to the lowest cost intervention (monthly phone calls). All randomized sites with outcome data will be included in the analysis, following the intent-to-treat principle. Organizational covariates in the analysis include program size, management score, and state.

**Discussion:**

The study offers seven recommendations for conducting a large-scale cluster-randomized trial: provide valuable services, have aims that are clear and important, seek powerful allies, understand the recruiting challenge, cultivate commitment, address turnover, and encourage rigor and flexibility.

**Trial Registration:**

ClinicalTrials. govNCT00934141

## Background

The field of addiction treatment serves only a small proportion of people who need its help. The 2009 *National Survey on Drug Use and Health *estimates that 23.5 million Americans age 12 and older needed treatment and 2.6 million received treatment from addiction treatment facilities [1]. Moreover, only about one-half of people who start treatment complete it [2,3], suggesting that about 6% of people who needed treatment in 2009 completed it-and that the addiction treatment system can substantially improve. That system (of addiction treatment facilities) is the subject of this study (not addiction treatment delivered in primary care, self-help, or faith-based organizations). The focus is systems- rather than client-level factors that relate to the effectiveness of treatment.

Several barriers stand in the way of people getting treatment. Individuals who call a program for help often encounter problems such as complicated admission systems, poorly designed phone systems, and a rude reception [4]. As a result, many who call do not make an intake appointment. More than one-half of those who do make intake appointments fail to attend [5,6]. Appointment delays, waiting lists, and requests that clients call back defer admission, discourage early engagement, and lead to appointment cancellations and no-shows, a low retention rate, and poor outcomes [7]. While these hurt the patient, they also contribute to inefficient capacity use. Providing an initial assessment soon after first contact greatly increases the chance of retaining clients [8,9]. The literature offers empirically tested strategies to improve client access and speed to treatment, such as taking walk-in clients [10], scheduling clients to come in within 24 to 48 hours of their first calling [11-13], and streamlining the intake process by reducing paperwork and other administrative barriers [14].

Just as several barriers impede access to treatment, so do various factors reduce retention. Clients most often leave treatment because of inconvenient treatment services [15], dissatisfaction with treatment [16], and family and work responsibilities [17]. Keeping clients in treatment is important because longer time in treatment relates to better outcomes [9,18,19] and completing treatment reduces addiction-related illnesses [20,21], crime [22], and joblessness [23].

Program leaders set organizational policies and processes that affect all of their clients, and they have the ability to change them. Given that the literature provides solutions for the access and retention problems in addiction treatment, what is the best way to disseminate improvements? Here, the literature provides surprisingly scant information [24]. One way to speed dissemination is to reduce organizational and process barriers using quality improvement (QI). Studies of organizational change, including QI, are difficult to do well. In particular, randomized controlled trials (RCTs) are difficult to conduct compared to studies of drug-effectiveness or other person-centered research for several reasons:

1. Sample size is a frequent limitation. Because randomization and analysis usually happens at the organizational rather than individual level, randomized trials of organizational change require a large number of organizations. Compared to individuals in clinical trials, it can be difficult to get organizations to participate, stay involved, and pursue the same aims.

2. Organizations differ and interventions, if they are to succeed, need to be tailored to the organizations. Differences in organizations make it hard to specify treatment as usual at the start of a study, so the researcher needs another baseline.

3. Participating organizations may be only those that volunteer and therefore not be representative of organizations in the field.

4. The intervention is hard to hold constant. In drug trials, the same pill is given to every subject in the intervention. But if, for instance, the intervention includes consulting, it is hard to hold consultation characteristics constant.

5. The environment is also hard to hold constant in studies that take months or years to complete.

The complexities of organizational change explain, in part, two criticisms often made of QI studies: the majority of QI efforts involve single organizations and a simple 'before and after' analysis, and QI efforts are multifaceted and studies of QI do not determine the active ingredients [25]. The scale and methods used in the study reported here are intended to respond to these criticisms.

Improvement collaboratives are one tool commonly used to deliver QI to organizations. Improvement collaboratives involve multiple organizations working together [26] to make processes better [27-30]. Collaboratives have produced disparate results in organizational change, from very positive [31-34], to mixed [30,35,36], to no effect [37]. This disparity may result, in part, from the methodological problems described above.

The study described here uses improvement collaboratives along with previously described principles and techniques developed by NIATx, formerly the Network for the Improvement of Addiction Treatment [38,39]. Like previous NIATx studies, this one (called NIATx 200) involved multiple organizations; unlike others, NIATx 200 involved randomizing the organizations using a cluster-randomized study design.

## Methods and design

### Study objectives and hypotheses

The primary research question of NIATx 200 is to determine which of four collaborative service combinations produces the greatest improvement in waiting time (days to treatment from first contact), rates of admissions to treatment, and continuation in treatment. The secondary research question is: What is the impact of the study interventions on treatment completion rates, the level of adoption and sustainability of the recommended practices, organizational readiness to adopt and sustain the new practices, voluntary employee turnover, and program margin? Interventions were delivered to and outcome analyses are performed at the program level.

We are also examining the cost of delivering each combination of services. The intent of the cost analysis is to assess the cost of disseminating different QI methods to state agencies that regulate substance abuse treatment. Our budget did not allow us to assess the cost to the treatment programs of implementing the services.

**Figure 1:**
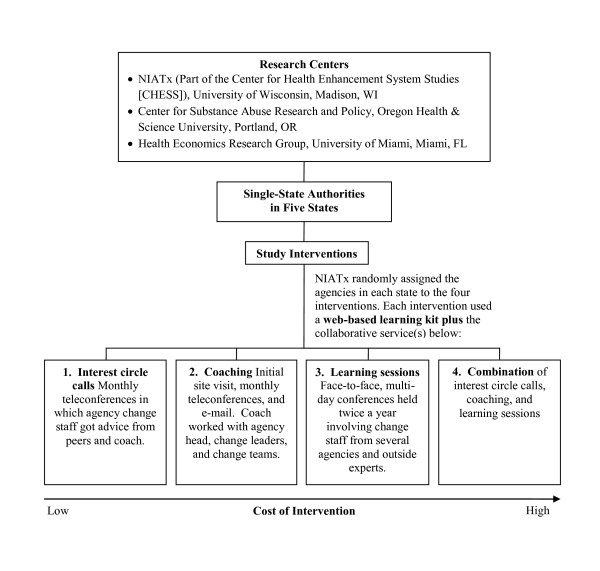
**The organization and design of the NIATx 200 cluster-randomized trial**.

NIATx 200 is a cluster-randomized trial with four interventions: interest circle calls, coaching, learning sessions, and the combination of interest circle calls, coaching, and learning sessions. Figure 1 shows the study's organization and design.

Interest circle calls are regularly scheduled (monthly in our case) teleconferences in which change team members from different programs discuss issues and progress and get advice from experts and one another. Interest circle calls are inexpensive and provide a regular meeting time for members to continue collaborating. If interest circle calls produce QIs, then change can be made widely at relatively low cost. But the quality of interest circle calls may vary by facilitator, and because the teleconferences are scheduled at particular times, they sometimes compete with other priorities, limiting participation. Although participants can listen to recordings of calls they miss, they lose the chance to interact.

Coaching assigns a process improvement expert to work with program leaders and change teams to help them make and sustain process improvements. In our study, coaching involved one site visit, monthly phone conferences, and e-mail correspondence. Coaches give a program ongoing access to an expert who tailors advice to the program and makes contacts with other experts and programs that have already addressed the same issue. However, coaching is expensive, and the match between program and coach may not be ideal. Program staff may also miss the camaraderie that comes from learning sessions and interest circle calls. Just as facilitator quality in interest circle calls may vary, so may the quality of coaching.

Learning sessions occur periodically-in our case, twice a year. These face-to-face multi-day conferences brought together program change teams to learn and gather support from outside experts and one another. Participants learn what changes to make and develop skills in QI (*e.g.*, creating business cases for improvements). Learning sessions raise interest in making changes and provide opportunities for program staff to share plans and progress. The sessions promote peer learning, increase accountability and competition, and give program staff the time to focus and plan as a team without distraction. Learning sessions also are costly, and the knowledge and excitement they produce can fade.

A common form of learning collaborative involves a combination of these services and is the final study intervention. A combination of interest circle calls, coaching, and learning sessions provides continuity and reinforcement over time and offers options for the way program staff can give and receive help. One would assume that this intervention would have the greatest effectiveness. But the combination is expensive and risks delivering inconsistent messages from different leaders and facilitators. The combination also risks exerting too much external pressure, thus reducing intrinsic motivation.

The protocol called for all four interventions to have the same goals during the three six-month intervention periods. For example, each program concentrated on reducing waiting time during months 1-6 of their involvement. During each intervention period, programs chose which practices to implement from those shown in Table 1. All interventions had access to the same web-based learning kit, which contained specific steps to follow, tools (*e.g.*, the walk-through, flow charting), ways to measure change, case studies, and other features. What varied were the methods of instruction and support that were wrapped around the learning kit: interest circle calls, coaching, learning sessions, or the combination of all three.

**Table 1:** NIATx 200 aims and promising practices

Reduce Waiting Time (months 1 to 6)	Increase Continuation (months 7 to 12)	Increase Admissions (months 13 to 18)
**Increase availability of time slots** • Add groups • Add time slots • Combine intake appointments • Double-book time slots **Increase the amount of counseling** • Ask clients to complete paperwork • Assign backup counselors • Cross-train counselors • Eliminate excessive paperwork • Reassign tasks • Transition existing clients **Eliminate appointments** • Establish walk-in hours • Provide interim services **Make appointments immediately** • Make appointments at the front desk • Make appointments during the first call for service • Open access to all time slots • Suspend financial arrangements	**Make it easy to enter treatment** • Connect with clients during first contact • Establish clear two-way expectations • Help eliminate barriers to treatment • Include family and friends • Offer an inviting physical environment • Remind clients about the next appointment **Make it difficult to refuse or quit treatment** • Collaborate with referral sources • Follow up with no-shows • Identify clients at risk for leaving and intervene **Make it easy to stay in treatment** • Assign peer buddies • Build community among clients • Have clients help create their treatment plans • Have clients select groups • Use contingency management	**Clients** • Offer a tour guide • Overlap levels of care • Blend levels of care • Include family and friends in discharge and admission planning • Use motivational interviewing • Use video conferencing • Map out continuing treatment • Orient clients to outpatient treatment • Offer telephone support • Reward attendance at the first outpatient appointment **Referrers** • Assign one contact person to each referral source • Schedule outpatient appointments before clients leave • Guide referral sources to make appropriate referrals • Tailor brochures for each referral source • Hold joint staffing • Streamline paperwork • Increase referral sources

### Ethics

The study received approval from the institutional review boards at the University of Wisconsin-Madison and Oregon Health and Science University and is registered at ClinicalTrials.gov (NCT00996645).

### Research team and study sites

The Center for Health Enhancement Systems Studies (CHESS), located at the University of Wisconsin-Madison, is a multidisciplinary team addressing organizational change to improve healthcare. NIATx is part of CHESS. The Center for Substance Abuse Research and Policy (at Oregon Health and Science University) works at the nexus of policy, practice, and research for the treatment of alcohol and drug dependence to improve evidence-based addiction treatment. The Health Economics Research Group (HERG) at the University of Miami conducts research on the economics of substance abuse treatment and prevention, HIV/AIDS, criminal justice programs, and health system changes. This core research team worked with five states through each state's single state agency (SSA) (the authority for substance-abuse treatment at the state level).

SSA administrative systems identified eligible programs. To be eligible to participate in NIATx 200, programs had to have at least 60 admissions per year to outpatient or intensive outpatient levels of care as defined by the American Society of Addiction Medicine (ASAM) and have received some public funding in the past year. Programs that had worked with NIATx in the past were excluded from participating. (Before the start of this study, a number of programs-fewer than 100 nationwide-had worked with NIATx.) All clients treated within an eligible program were deemed eligible and included in the analysis. SSAs and state provider associations promoted the study and helped recruit participants. Before randomization, NIATx assessed programs' readiness for change and management strength, and asked about which other treatment programs most influenced their own operations. Then programs were randomized to study interventions.

### Study measures

NIATx 200 has three primary outcomes: waiting time (days from first contact to first treatment), annual program admissions, and continuation in treatment through the first four treatment sessions. Data for the waiting time and continuation outcomes come from patient information collected, aggregated, and sent in by the SSAs at approximately 9, 18, and 27 months after the start of the intervention. Annual program admissions and other secondary outcomes (including voluntary employee turnover, treatment completion, and operating margin) were collected through surveys of executive directors conducted at baseline, mid-intervention, and project completion. The research team also surveyed staff members at the treatment programs and is using other measures, described below, to understand the role of mediators, moderators, and other factors that contribute to an organization's success in making changes. As others have demonstrated, QI efforts are much more likely to improve quality if they take place in a supportive context [40,41].

The Organizational Change Manager (OCM) measures an organization's readiness for change. Staff members at treatment programs completed the OCM. The OCM had good inter-rater reliability among respondents in field tests [42].

Organizations adopt many changes, but sustain few [43]. The 10-factor multi-attribute British National Health Services Sustainability Index is being used to predict and explain the sustainability of promising practices that programs implemented. A research trial involving 250 experts in healthcare policy and delivery, organizational change, and evaluation validated the model, which explained 61% of the variance in the sustainability of improvement projects [43].

The management survey measured 14 management practices at the beginning of the study. The survey was based on the instrument developed by Bloom and Van Reenan [44]. The published results indicate that good management practice is associated with shorter waiting time, weakly associated with revenues per employee, and not correlated with operating margins. Better management practices were more prevalent in programs with a higher number of competitors in the catchment area [45].

The Drug Abuse Treatment Cost Analysis Program (DATCAP) is a data-collection instrument and interview guide that measures both direct expenses and opportunity costs. Although DATCAP was initially used in the field of drug abuse treatment, the instrument is now used in treatment programs in many social-service settings. DATCAP was modified for this study to capture the economic costs to an SSA of developing and providing services [46].

### Sample size

Power calculations were predicated on the idea that the unit of analysis is the program rather than the client. Power was calculated for various sample sizes, with consideration given to anticipated recruitment levels in each state. An attrition rate of 20% was assumed in the sample size calculations. It was determined that a sample size of 200 programs would provide 80% power to detect a difference of 3.2 days in waiting time, 7.5% difference in continuation, and 14.2% difference in the log of admissions with alpha = 0.05. These levels of improvement for each outcome were deemed to be clinically or organizationally meaningful.

### Randomization

The study design calls for nesting of agencies within states; as such, randomization of programs took place state by state. Though recruitment took place over a period of several months, all programs were randomized at a single point in time at the end of the recruitment period in each state. The randomization was stratified by program size and a quality-of-management score generated during a baseline interview with program leaders [45]. The project statistician generated the allocation sequence. The University of Wisconsin research team enrolled participants and assigned participants to interventions. Assignments to interventions were made using a computerized random number generator. Multiple programs within the same organization were assigned to the same intervention to avoid contamination. Neither the participants nor the study team were blind to the assignments.

### Timeline

The five states participating in NIATx 200 were divided into two cohorts. Cohort one had three states; cohort two had two states. For each cohort, randomization took place in the two to three weeks before the first six-month intervention began. See Table 2. Baseline data were gathered in a period of up to three months before randomization.

**Table 2:** NIATx 200 Timeline

Cohort	Reduce Waiting Time				Increase Continuation				Increase Admissions			
	
	Intervention (6 mo.)		Sustain (9 mo.)		Intervention (6 mo.)		Sustain (9 mo.)		Intervention (6 mo.)		Sustain (5 to 8 mo.)*	
	
	Start	Stop	Start	Stop	Start	Stop	Start	Stop	Start	Stop	Start	Stop
1	10/07	3/08	4/08	1/09	4/08	10/08	11/08	8/09	11/08	3/09	5/09	12/09

2	2/08	7/08	8/08	4/09	8/08	1/09	2/09	10/09	2/09	7/09	8/09	12/09

Start dates = first day of each month indicated.

Stop dates = last day of each month indicated.

*The sustainability period for the third intervention was shorter than for the other interventions because data collection ended in December 2009.

### Procedures

The state authorities recruited programs to participate in NIATx 200. They promoted the study at meetings and by word of mouth, and wrote letters to the CEOs of programs. They also conducted meetings so the leaders of eligible programs could learn more about the study. At these meetings, the research team and SSA directors explained that programs would use one of four methods to improve processes that affect access and retention. Peer programs with NIATx experience explained the improvements they had made using the same methods and showed changes in data that resulted from the improvements. SSA directors outlined the benefits and responsibilities of programs in the study. Programs would gather pretest data, be randomly assigned to one of four study interventions, and receive 18 months of support. During months 1 to 6, they would focus on reducing waiting time; in months 7 to 12, increasing continuation rates; and in months 13 to 18, increasing admissions. Afterward, the program could join state-led activities to sustain changes. The SSA would send client data to the researchers about waiting time, admissions, continuation, and treatment completion. The program CEO would name from the staff an influential change leader. The leader and staff at each program would complete surveys during the pretest period and at 9, 18, and 27 months. These surveys addressed employee turnover, new practices initiated, number of employees, revenue, and operating margin. Staff would complete surveys about how the program makes and sustains changes. The program would receive minimal compensation for reporting these data.

### Data analysis

The treatment program comprises both the unit of randomization and the unit of analysis in the study. For the primary analysis, the protocol calls for us to aggregate client records to compute monthly averages for each program's waiting time and continuation rates. The unit of analysis will be a vector of program-month results based on these aggregated values. We will fit a mixed-effect regression model to these monthly observations, including terms to isolate state and intervention effects. We are aggregating admissions to a yearly level to compensate for seasonality. We will use random effects to model the correlation among outcomes from the same program. Organization-level random effects will be included to model the correlation among programs within the same organization. Interventions will be ordered by the cost of implementation and compared in a pair-wise fashion to the lowest cost intervention (interest circle calls). All randomized programs with available outcome data will be included in the analysis according to the intent-to-treat principle. The analysis will be conducted by originally assigned intervention regardless of how much programs participated in the learning services. Organizational covariates accounted for in the analysis will include program size, management score, and state affiliation.

### Illustration of a program participating in NIATx 200

The following scenario shows what a program assigned to the coaching intervention might have experienced. Assignment to the coaching intervention meant that the program had a process improvement coach visit at the beginning of the study, call every month for the next 18 months, and communicate via e-mail.

A program began its work once the CEO named a change leader and change team. This group learned from the research team in the first week of their participation how to do a walk-through, which is a tool that shows staff members what it is like to experience dealing with the program as a client does. The program also had a coach assigned to it by the research team. The coach phoned the CEO, change leader, and change team members to introduce herself and set an agenda for a site visit. The coach also reviewed with the change team the results of the walk-through, an experience that revealed many issues to the change team, including long waiting times. The coach also encouraged change team members to examine case studies on the study website.

The site visit allowed the coach and change team members to get to know each other. The coach explained evidence-based improvement principles to the change team and different ways of reducing waiting time, the goal of the study in the first six months. The change team had to decide on one change to make first to reach this goal. In this example, the program decided to adopt a change they learned about from a case study on the study website: Eliminate appointments and instead invite callers to walk in the next morning, complete intake and assessment, and start treatment by noon. Among other things, the change team learned that making this change had resulted in the outpatient program's significantly increasing its revenue. Using information from the case study, the coach helped the change team review in detail how the program made this change-what data they collected, what steps they took, and what protocols they used to train staff for handling the high volume of walk-ins. Finally, the coach helped the change team figure out how to collect pretest data and start the rapid-cycle change process. In two weeks, the program would have enough pretest data (on about 25 clients) to start the change process.

Once pretest data were collected, the first rapid-cycle change (or plan-do-study-act cycle) began. The first three callers on Monday were invited to come anytime the next morning to be seen right away. Two callers jumped at the chance. One had to work but offered to come after work. The change leader (who was the program's medical director) did the intake and assessment with one client to experience the new process. The clinician did the same with the other client. At the end of the day, the medical director and clinician modified the change to allow walk-ins at the end of the day too. The next version of the change started Thursday and involved the first five people who called and two clinicians. After this change, the staff identified additional concerns and made other adaptations. Throughout the rapid-cycle changes, the change leader worked side-by-side with clinical staff (a key part of the strategy) to understand problems and make modifications. After staff discussions, the group decided on one last change. Now the new process would be tested with anyone who called for the next week (as medical director, the change leader had the authority to implement changes). In the space of three weeks, the overall goal of taking walk-ins was achieved by making and adapting small changes several times, until the process worked well. As a result, the program began serving more clients without adding staff.

During the remainder of the six months when the goal was to reduce time to treatment, the program continued to introduce and refine other changes (see the possibilities in Table 1), using the rapid-cycle change model. Starting in month seven, the goal changed to increasing continuation in treatment. The program did another walk-through to initiate a new series of changes to achieve this goal.

## Discussion

### Lessons learned

Seven important lessons from conducting NIATx 200 are described below.

### Provide services that participants value

The states and programs in this study believed they were participating in an important study and getting services that were valuable. One reason for this and a tool that helped in recruitment was presenting the business case for process improvement-showing eligible treatment programs the financial benefits that other programs gained as a result of adopting the NIATx practices used in the study.

### Have clear and important aims and practices

Research established and tested the goals and practices of the NIATx model (see Table 1) before the study started [38]. As a result, the goals and practices were expressed clearly for the intended audience and were embraced as important. In addition, for this study, the research team and others in addiction treatment laid out a 'road map' of activities, objectives, and competencies for each phase of the study. This document, along with the web-based learning kit, gave everyone in the study a common set of information. Additionally, the goals were known and understood by researchers, coaches, the states, interest-circle facilitators, and program staff.

### Seek powerful allies to recruit and retain organizations

The research team formed partnerships with state authorities for substance abuse treatment to recruit programs to the study and collect and report data. The work of the SSAs proved critical to the success of the project because they had the systems, relationships, and leverage essential to the task. The SSAs used their data systems to identify eligible programs and facilitate data collection. Most important, through promotion, personal contacts, and incentives, the SSAs encouraged programs to sign up for and participate in the study.

### Understand the challenge of recruiting organizations

When we started the study, we recruited about 30 programs in each state fairly easily. After this, it was difficult to recruit more programs. Programs in the first group in each state were interested in change; programs recruited later were less enthusiastic. This experience suggests that studies of organizational change can probably recruit most effectively from the approximately 48% of organizations in the categories that Rogers calls 'early adopters' and 'early majority'-and that it is difficult to compel other organizations to take part [47]. This recruitment challenge is likely to present itself in all studies of organizations, especially when organizations volunteer for the study and are not part of one larger group under a single authority. To improve generalizability, it is important to select target organizations and work very hard to convince them to join.

### Cultivate commitment and engagement throughout the study

Once organizations signed up for the study, it was easy for people's initial enthusiasm to fade. Our team worked with the states throughout the study to retain buy-in and participation. This meant conducting phone-based meetings with the SSAs twice a month for more than two years, starting before recruitment took place, to involve them and seek their advice in all levels of planning and executing the study. Researchers invited state and program representatives to attend when they made presentations at conferences to acknowledge their importance in the work. The SSAs gave technical assistance to programs to help them provide the data required by the study. State leaders also sent letters of encouragement to programs; attended learning sessions to learn and set an example; hosted, with the research team, state-specific calls for programs; and, at statewide meetings, recognized programs participating in the study and invited them to tell their success stories.

### Address turnover

Staff turnover presents another ongoing challenge in long-term studies of organizational change. The loss of a staff member who acts as a contact point for data collection or project management can be very disruptive to a program's participation in the study. Teamwork can address this problem. For example, three people were involved in the project from each SSA and treatment program and participated in calls and meetings-an executive, the change leader, and a data coordinator. If one person left his job, the other two could pitch in and, when a replacement was selected, help that person learn the history and her role going forward.

### Encourage both rigor and flexibility

This study, like all large organizational interventions, required both rigor and flexibility, a need for clarity and a tolerance of ambiguity, sticking to the plan and making adjustments to it. The unforeseen will arise-and sometimes the study benefits from it. For example, programs struggled at first to supply data about the time between a prospective client's first call and getting into treatment. Before the study, most programs did not report this measure and the states' data systems had to be modified to include it. As a means of validating these data, research staff called each program once a month for the length of the study to obtain data about the days between first contact and getting into treatment. The information gained from these more than 6,600 calls provides a rich fund of other data to analyze. For example, some insured clients seeking treatment wait longer to get into treatment than uninsured clients, a finding that bears further investigation.

### Limitations and challenges

Randomized trials of organizational change always pose challenges. NIATx 200 has two notable limitations.

### Including a control and blinding

The gold standard in evaluation research is the randomized, placebo-controlled, double-blinded clinical trial [48]. Conceptualizing a 'placebo' in this study of organizational change proved challenging. Given that monthly teleconferences were available to the general public through a separate NIATx initiative, the interest-circle-call intervention functioned as the control in this study. Double blinding is logistically impossible. It would be impractical, for example, to plan a learning session without knowing which programs to register at the conference hotel. Further, blinding participants would be antithetical to the collaborative nature of interest-circle calls and learning sessions. Within each intervention, participants were encouraged to learn from one another as part of the collaborative.

### Intervention and research costs

In developing the proposal for this work, we realized we would need 200 programs (about 50 programs per intervention) to have enough power to produce reliable results. This determination gave us a sense of the scale and cost of NIATx 200. We recognize that such a study is costly to implement and evaluate, and it will be hard to replicate at a similar scale. It will, however, provide extensive data for the analysis of organizational change for years to come.

## Conclusion

NIATx 200 advances organizational change and QI in several important ways. First, the findings will provide quantitative evidence of whether QI can improve fundamentally important elements of addiction treatment (*i.e*., access and retention). Second, the sheer scope of the study (201 treatment programs nested within five states) is unprecedented in QI research, and will provide insights on the effectiveness as well as the cost of delivering an organizational change intervention across multiple organizations. Third, in contrast to research that has examined the effects of a single tool or strategy (such as a checklist), NIATx 200 will advance the field by identifying the active ingredients in a multi-factorial QI strategy. Distance learning, coaching, and learning sessions have been key elements in many QI initiatives. Despite their widespread use, few rigorous studies have estimated their effectiveness. NIATx 200 provides a unique and specific test of their relative contributions to improvement.

The study examines methods for moving process improvement from a small number of organizations led by a national office to a state-run initiative involving many programs. Qualitative data will show the client experience in seeking care. Because states had to develop new data systems (*e.g.*, to collect and report date of first contact), the study will provide insights about how states manage data systems and how they are prepared to address information system-related issues in healthcare reform. The study also collected information on which treatment programs exert influence over others, making it possible to examine influence characteristics in addiction treatment and how these may affect the dissemination of change.

It may also be possible to identify practices of organizations that are willing to make changes. The study will describe these practices in detail, assess the impact of the practices on clients, and give practical advice about how to implement them. The measures of management quality, readiness for organizational change, and the potential for sustainability allow validation of those instruments and further understanding of the conditions that contribute to lasting organizational change.

The data also will allow for detailed studies of moderation. What type of programs benefit from which QI approach? What type of coaching works best with which types of programs? Under what conditions do specific promising practices have the greatest success? One key product of the study will be the resources developed to teach QIs. For example, in preparing for the study, many promising practices were described in 'how to' manuals. These manuals are available to organizations interested in learning about and using these QI techniques. The diverse, real-world results of the study should provide direction for state leaders, heads of programs, and others about how best to improve access to and retention in treatment.

Finally, NIATx 200 shows that rigorous randomized trials of organizational change can be designed and conducted. The information produced will expand our understanding of treatment organizations and provide stakeholders in healthcare delivery a body of research they need to move our healthcare systems forward.

## Competing interests

The authors declare that they have no competing interests.

## Authors' contributions

DG and DM designed the study. AQ, JF, and AP contributed to the design and managed the acquisition of data and the implementation of the project. MF and KM led the economic design. AQ drafted the original manuscript. All authors read and approved the final manuscript.
